# Widespread use of the “ascidian” mitochondrial genetic code in tunicates

**DOI:** 10.12688/f1000research.21551.2

**Published:** 2020-04-14

**Authors:** Julien Pichon, Nicholas M. Luscombe, Charles Plessy

**Affiliations:** 1 Genomics and Regulatory Systems Unit, Okinawa Institute of Science and Technology Graduate University, Onna-son, Okinawa, 904-0495, Japan; 2 Université de Paris, Paris, France; 3 The Francis Crick Institute, London, NW1 1AT, UK; 4 Genetics Institute, University College London, London, WC1E 6BT, UK

**Keywords:** Tunicate, Oikopleura, Genetic code, Mitochondria, Cytochrome oxidase subunit I

## Abstract

**Background:** Ascidians, a tunicate class, use a mitochondrial genetic code that is distinct from vertebrates and other invertebrates. Though it has been used to translate the coding sequences from other tunicate species on a case-by-case basis, it is has not been investigated whether this can be done systematically. This is an important because a) some tunicate mitochondrial sequences are currently translated with the invertebrate code by repositories such as NCBI GenBank, and b) uncertainties about the genetic code to use can complicate or introduce errors in phylogenetic studies based on translated mitochondrial protein sequences.

**Methods:** We collected publicly available nucleotide sequences for non-ascidian tunicates including appendicularians such as Oikopleura dioica, translated them using the ascidian mitochondrial code, and built multiple sequence alignments covering all tunicate classes.

**Results: **All tunicates studied here appear to translate AGR codons to glycine instead of serine (invertebrates) or as a stop codon (vertebrates), as initially described in ascidians. Among Oikopleuridae, we suggest further possible changes in the use of the ATA (Ile → Met) and TGA (Trp → Arg) codons.

**Conclusions:** We recommend using the ascidian mitochondrial code in automatic translation pipelines of mitochondrial sequences for all tunicates. Further investigation is required for additional species-specific differences.

## Introduction

Tunicates are marine animals that have acquired the capacity to produce cellulose by horizontal gene transfer approximately 500 million years ago ( Matthysse *et al.*, 2004; Nakashima *et al.*, 2004). Together with vertebrates and cephalochordates, they belong to the chordate phylum, in which they share morphological features such as a muscular tail during larval stages. Phylogenetic studies place the tunicates as the closest living relatives of vertebrates ( Delsuc *et al.*, 2006). Tunicates can be subdivided in three classes: Thaliacea (free-swimming colonial species, for instance salps or dolioids), Appendicularia (free-swimming solitary species with an adult morphologically similar to the larval stage of other tunicates), and Ascidiacea (attached to solid substrates in their adult stage, for instance sea squirts). The relationship between these classes and therefore their mono- or paraphyly has been revised multiple times. For instance the 18S rRNA analysis of Stach & Turbeville (2002) nested Appendicularia within Ascidiacea, but more recently Delsuc *et al.* (2018) placed them as sister groups using a multigene approach. The paraphyly of Ascidiacea is now widely accepted, as the above studies and others demonstrated that they contain the Thaliacea.

Mitochondrial genomes undergo major changes at the geological time scale due to their small size and clonal reproduction, including changes to their genetic code ( Osawa *et al.*, 1992). In animals, alternative genetic codes have first been found in large clades, for instance echinoderms ( Himeno *et al*., 1987) and hemichordates ( Castresana *et al*., 1998), but more recent works underline the presence of changes deeper in the phylogenetic tree, for instance within nematodes ( Jacob *et al*., 2009) and within hemichordates ( Li *et al*., 2019). The first evidence that ascidians use a specific mitochondrial genetic code stemmed from observations that the cytochrome c oxidase subunit 1 ( *Cox1*) sequence from *Halocynthia roretzi* ( Yokobori *et al.*, 1993) and the *Cox3* sequence of *Pyura stolonifera* ( Durrheim *et al.*, 1993) are interrupted by stop codons if translated using the vertebrate mitochondrial code. Reassignment of AGR codons to glycine was later confirmed by the discovery of a glycine (Gly) tRNA in the *H. roretzi* genome ( Yokobori *et al.*, 1999) and by the sequencing of its anticodon (U*CU) ( Kondow *et al.*, 1999). Apart from the AGR codons, the ascidian code is similar to the vertebrate and the invertebrate ones, with ATA assigned to methionine (Met) and TGA to tryptophan (Trp) ( Yokobori *et al.*, 1993).

This genetic code is known as the “ascidian” genetic code; however, it is also used by non-ascidian tunicates, such as the thaliacean *Doliolum nationalis* ( Yokobori *et al.*, 2005). The possibility that this genetic code emerged earlier than tunicates was raised by the study of partial genome sequences of *Branchiostoma lanceolatum* ( Delarbre *et al.*, 1997) leading to the proposition that AGR might encode Gly in cephalochordates. While this seemed to be supported by the discovery of a putative TCT (Gly) tRNA in the full mitochondrial genome of *B. lanceolatum* ( Spruyt *et al.*, 1998), this hypothesis was later ruled out by an analysis of the related amphioxus *Branchiostoma floridae* ( Boore *et al.*, 1999), and has not been reconsidered since. Finally, studies on the appendicularian branch showed compatibility between the mitochondrial sequence of *Oikopleura dioica* and the ascidian code ( Denoeud *et al.*, 2010). Nevertheless, support for compatibility was not demonstrated explicitly for the ATA and TGA codons and the mitochondrial sequence of *O. dioica* were not released in International Nucleotide Sequence Database Collaborations (INSDC) databanks.

Cox1 is the most conserved mitochondrial protein. Although no mitochondrial genome has been fully sequenced yet for appendicularians, partial *Cox1* sequences are present in the INSDC databanks for Oikopleuridae. Sakaguchi *et al.* (2017) reported that all *Oikopleura* mitochondrial sequences ( AY116609– AY116611 and KF977307) may be contaminations from bacteria or cnidarians, and provided partial sequences for *Oikopleura longicauda* in the same study. Partial mitochondrial sequences were published for *Bathochordaeus* and *Mesochordaeus* species by Sherlock *et al.* (2017). In addition, Naville *et al.* (2019) recently published draft genome for several appendicularian species. Therefore, to assess whether the ascidian mitochondrial code is used across the whole tunicate subphylum, we took advantage of these public data and prepared a curated alignment of Cox1 sequences comprising representatives of the major tunicate branches, to study the consensus sequences at conserved residues.

## Methods

We identified *Cox1* and Cytochrome b ( *Cob*) gene sequences for *Oikopleura longicauda*, *Mesochordaeus erythrocephalus* and *Bathochordaeus stygius* by screening published genome assemblies ( Naville *et al.*, 2019) with the partial Cox1 sequence of *O. longicauda* LC222754.1 ( Sakaguchi *et al.*, 2017) using tblastn and the ascidian mitochondrial code ( -db_gencode=13) ( Gertz *et al.*, 2006). Mitochondrial genome sequences were then translated using the cons and getorf commands from EMBOSS ( Rice *et al.*, 2000), using the ascidian mitochondrial code.

### *Oikopleura longicauda* 

We identified the circular contig SCLD01101138.1 (length: 10,324 nt) as a potential mitochondrial genome, and translated *Cox1* from position 4530 to 6230. We also translated *Cob* from 3697 to 4668.

### *Mesochordaeus erythrocephalus* 

We translated *Cox1* in contig SCLF01725989.1 (length 7,034 nt) on reverse strand from position 1792 to 272. Using the same method with *O. longicauda*’s Cob sequence as a bait, we also recovered a *Cob* sequence from contig SCLF01109548.1 (length 5,010 nt), reverse strand, 1604 to 2590.

### *Bathochordaeus stygius* 

We used the consensus of the published *B. stygius Cox1* sequences KX599267.1 to KX599281.1 from GenBank ( Sherlock *et al.*, 2017), to screen the genome and scaffold SCLE01415711.1 (length 10,388 nt) gave a perfect hit. We translated *Cox1* from position 8054 to 6522 on the reverse strand, and a partial *Cob* sequence from scaffold SCLE01415711.1 (2319 to 2963, reverse strand). We also found a second fragment aligning well with C-terminal sequences between positions 2373 and 1978, but we did not include it due to the difficulty of resolving the overlap between both fragments. When screening with the *M. erythrocephalus Cox1* sequence recovered above, we found that another scaffold SCLE01416475.1 gave a perfect hit, hinting at a possible contamination.

### *Oikopleura dioica* 

To assemble a *Cox1* sequence in *O. dioica*, we downloaded expressed sequence tags (file 10_ESTall.txt) from Oikobase ( Danks *et al.*, 2012) and extracted hits matching the *O. longicauda* sequence using tblastn (see above). We then aligned and visualised the hits using Clustal Omega ( Sievers *et al.*, 2011) and SeaView ( Gouy *et al.*, 2009), filtering out those too short or introducing gap columns. Inspection of the alignment let us notice three possible haplotypes. We generated a consensus for each of them, translated them (see above) and trimmed the proteins sequences in order to match the length of the other reference sequences in the alignment. All variants found between the haplotypes were synonymous codons. We used the same methodology to generate a consensus for *Cob* and translate it.

### *Cox1* accession numbers

*Bathochordaeus charon* KT881544.1 ORF2 translated with ascidian code; *Bathochordaeus stygius*: SCLE01415711.1[8054:6522] translated with ascidian code; *Branchiostoma lanceolatum*: BAD93656.1; *Caenorhabditis elegans*: NP_006961.1; *Ciona intestinalis*: CAL23359.2; *Clavelina oblonga*: YP_009029840.1; *Doliolum nationalis*: BAD86512.1; *Halocynthia roretzi*: NP_038239.1; *Mesochordaeus erythrocephalus*: SCLF01725989.1[1915:260] translated with ascidian code; *Mus musculus*: NP_904330.1; *Oikopleura dioica*: consensus of Oikobase contigs (see file 10_ESTall.txt) KT0AAA24YA11, KT0AAA22YO17, KT0AAA22YO04, KT0AAA13YK14, KT0AAA18YK22, KT0AAA16YP04, KT0AAA13YE23, KT0AAA8YH10, KT0AAA4YK01, KT0AAA24YE23, KT0AAA18YO18, KT0AAA3YP19, KT0AAA10YF12; *O. longicauda*: SCLD01101138.1[4678:6230] translated with ascidian code; *Salpa thompsoni*: BBB04277.1.

### *Cob* accession numbers

*Bathochordaeus stygius*: SCLE01415711.1[2963:2319] translated with ascidian code; *Branchiostoma lanceolatum*: BAD93666.1; *Caenorhabditis elegans*: NP_006958.1; *Ciona intestinalis*: CAL23352.2; *Clavelina oblonga*: YP_009029843.1; *Doliolum nationalis*: BAD86520.1; *Halocynthia roretzi*: NP_038246.1; *Mesochordaeus erythrocephalus*: SCLF01109548.1[1604:2590] translated with ascidian code; *Mus musculus*: NP_904340.1; *Oikopleura dioica*: consensus of Oikobase contigs KT0AAA23YJ17, KT0AAA16YJ22, KT0AAA17YO14, KT0AAA10YI15, KT0AAA18YI18, KT0AAA11YF07, KT0AAA10YG05, KT0AAA1YH02, KT0AAA12YH10, KT0AAA12YC07, KT0AAA12YC07, KT0AAA18YM15 (see file 10_ESTall.txt); *O. longicauda*: SCLD01101138.1[3697:4668] translated with ascidian code; *Salpa thompsoni*: BBB04269.1.

### Sequence alignments

Translated *Cox1* and *Cob* sequences were aligned using Clustal Omega ( Sievers *et al.*, 2011) and SeaView ( Gouy *et al.*, 2009). The alignments were post-processed using the showalign -show=n command of EMBOSS ( Rice *et al.*, 2000) to show the differences to the inferred consensus. Graphical processing of the alignments were performed with Jalview ( Waterhouse *et al.*, 2009). The codon sequences encoding Cox1 and Cob of the tunicate species were then added aligned to the corresponding amino-acid (three lines per species, see *Extended data* ( Plessy & Pichon, 2019)) and then the text files were transposed, so that each line would correspond to a single position in the alignment, and interrogated with custom Unix commands to compute the tables presented in this manuscript.

## Results

### AGR encodes for Gly across all tunicates

**Figure 1.**
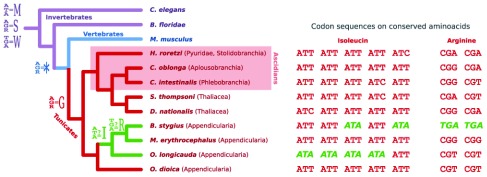
Left: Cladogram illustrating the relations between the species selected in study. Different branch colors indicate different mitochondrial genetic codes. Codon assignments with an equal sign indicate how the nucleotide sequences were translated. Codon assignments with a question mark indicate a possible finding, but were not used for translation. Ascidians, in which the AGR to Gly codon reassignment was initially discovered, are highlighted among the tunicates. Right: codon sequence of *Cox1* genes on positions where proposed changes of genetic code would make all species use the same amino acid.

**Figure 2.**
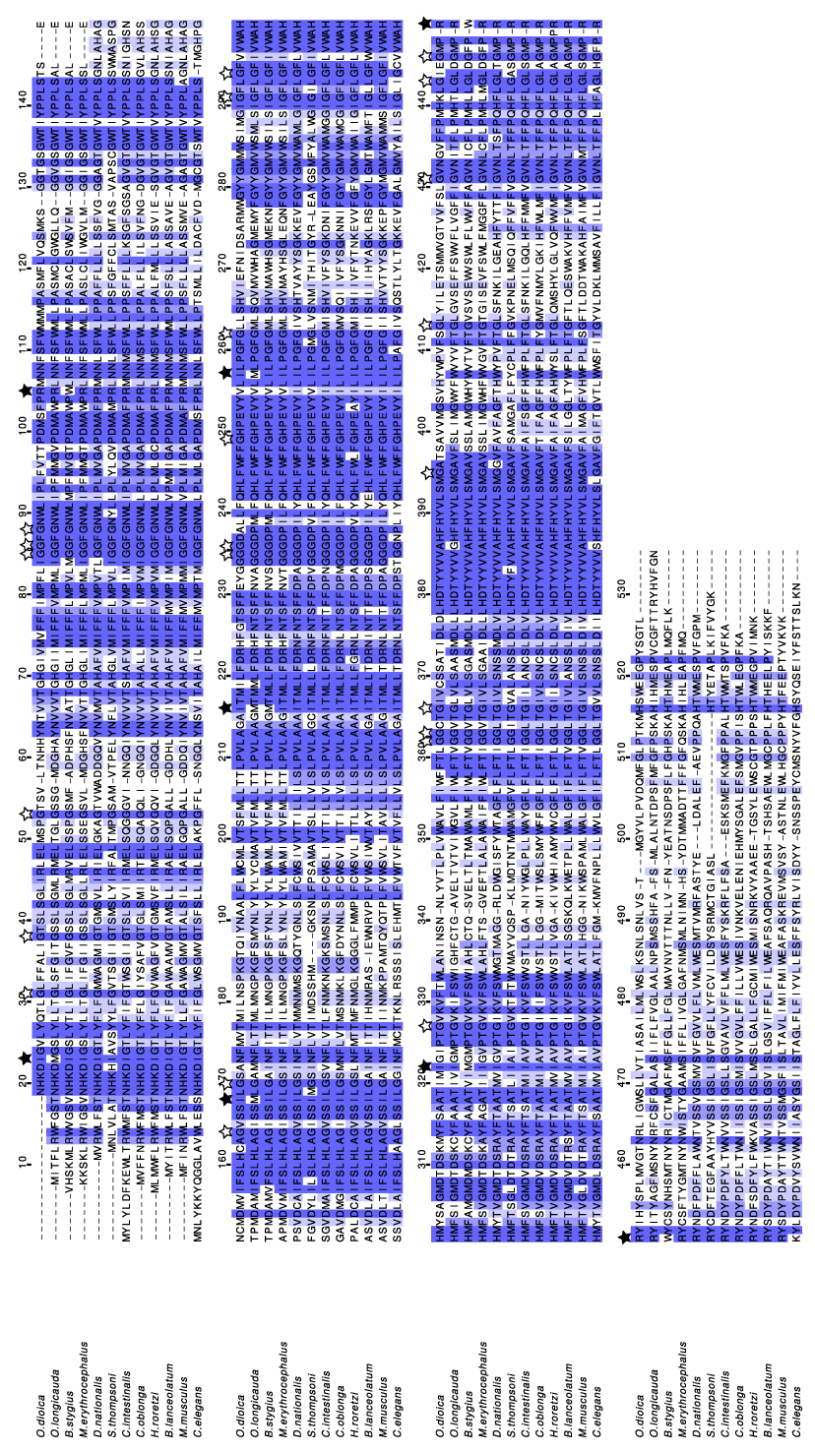
Sequence alignment of Cox1 proteins. White stars indicate conserved cysteines when at least one tunicate uses an AGR codon. Black stars indicate positions suggesting a different genetic code.

**Figure 3.**
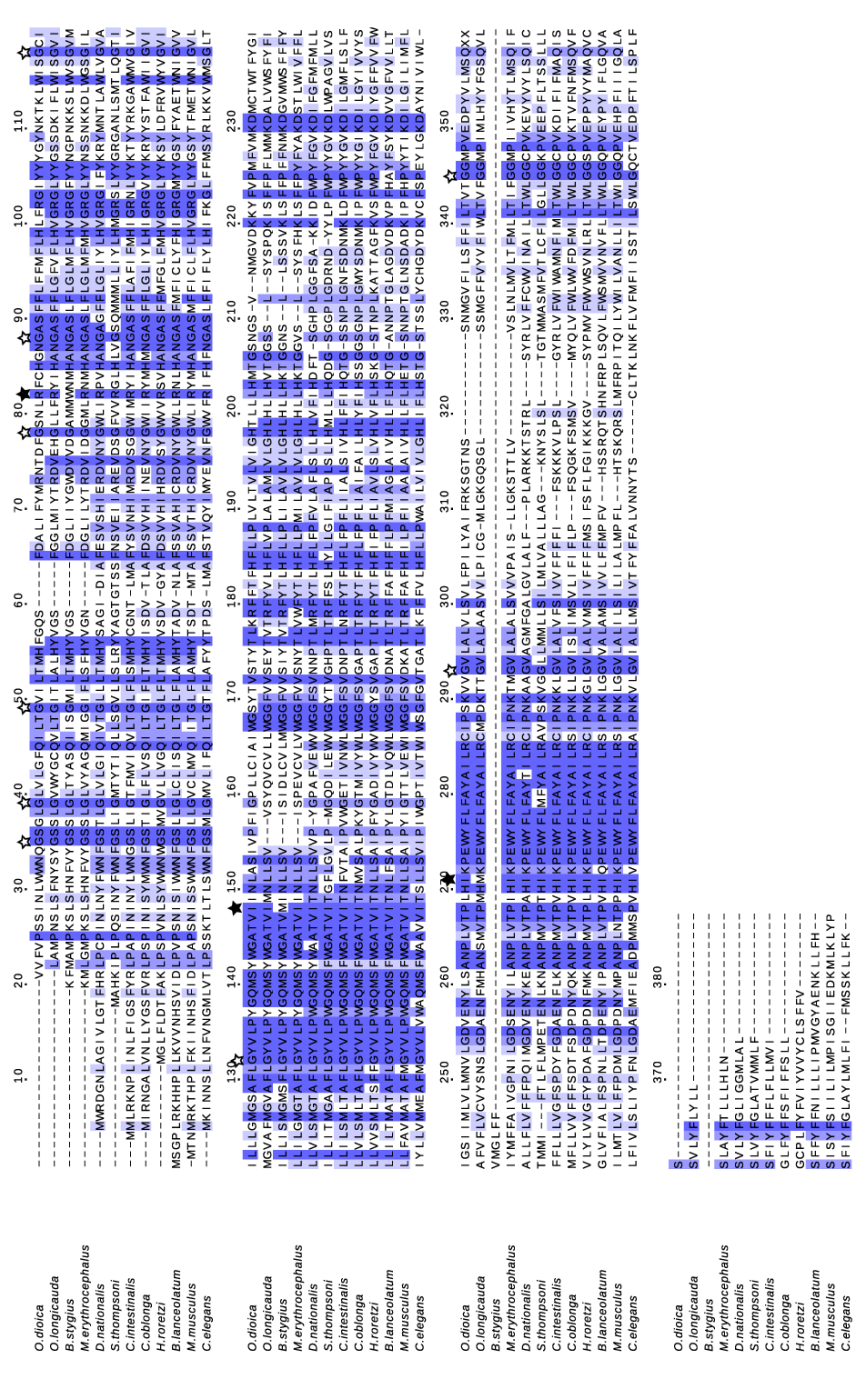
Sequence alignment of Cob proteins. White stars indicate conserved cysteines when at least one tunicate uses an AGR codon. Black stars indicate positions suggesting a different genetic code.

We selected species according to sequence availability and to ensure coverage of the tunicate subphylum in a way that stays broad under the various hypotheses of monophyly or paraphyly for its major groups. For ascidians, we have included the phlebobranchian *Ciona intestinalis*, the aplousobranchian *Clavelina oblonga* and the pyurid stolidobranchian *Halocynthia roretzi*. For thaliaceans, we selected *Doliolum nationalis* and *Salpa thompsoni*. For appendicularians we selected *Oikopleura dioica*, *Oikopleura longicauda*, *Bathochordaeus stygius* and *Mesochordaeus erythrocephalus*. We ensured that all tunicate sequences were translated with the ascidian mitochondrial genetic code. Lastly, we included outgroup sequences from *Caenorhabditis elegans* and *Branchiostoma lanceolatum* (invertebrate mitochondrial code) and from *Mus musculus* (vertebrate mitochondrial code) to better highlight conserved amino acid positions. In Figure 1, we illustrate the relation between these species based on the phylogeny of Naville *et al.* (2019) for appendicularians and of Delsuc *et al.* (2018) for the other tunicates. We prepared *Cox1* sequences from the selected species using mitochondrial genomes (for ascidians, thaliaceans, and outgroups), from draft genomes in which we found a putative mitochondrial contig after screening with a partial or a related *Cox1* sequence (for *O. longicauda*, *B. stygius*, and *M. erythrocephalus*) and from EST sequences (for *O. dioica*). We aligned the translated Cox1 and Cob sequences ( Figure 2 and Figure 3) and inspected the positions where all species use the same amino acid. Conserved glycines supported the use of AGR codons across the whole tunicate clade. We confirmed this observation with *Cob* sequences obtained with the same method.

### Possible lineage specific use of ATA Ile and TGA Arg codons

We then searched for positions where a single tunicate species differed from the other sequences with the same replacement amino acid more than once. We found multiple cases of methionine being replaced by isoleucine and arginine replaced by tryptophan in *O. longicauda* and *B. stygius* ( Figure 2). Given their phylogenetic proximity, we grouped the two species in the analysis below and we calculated the number of mismatches to the other sequences. We redefined a position as “conserved” if there is at most one mismatch from one sequence to the others.

*M. erythrocephalus* does not seem to use ATA codons and *O. longicauda* and *B. stygius* use ATA codons at positions where all other species had an isoleucine (Ile) ( Table 1 and Table 2). In the ancestral invertebrate mitochondrial code and the sister vertebrate code, ATA encodes Met. Although Met and Ile both have hydrophobic side chains that often can substitute for each other, this also suggests a change of the genetic code. Evidence for this is that 1) non-appendicularian species do not display ATA codons at positions where all other species encode Ile; 2) the change would be parsimonious as *O. longicauda*, *B. stygius* and *M. erythrocephalus* are more closely related to each other than to *O. dioica* ( Naville *et al.*, 2019); and 3) these three species never have ATA codons at positions where Met is conserved in every species (in contrast to *O. dioica*). Furthermore, reversion of the ATA codon to Ile have occurred in other branches of the tree of Life, for instance in echinoderms ( Jacobs *et al.*, 1988). Finally, inspection of a partial *Cox1* sequence of the related *Bathochordaeus charon* (KT881544.1) provided one extra instance of an ATA codon at a conserved Ile position.

**Table 1.** ATN codons in *Cox1* in Oikopleuridae.

	*O. dio.*	*O. lon.*	*B. sty.*	*M. ery.*
ATA number	22	12	16	0
ATC number	3	8	0	2
ATG number	14	34	25	33
ATT number	29	18	21	38
ATA on cons. Met	5	0	0	0
ATA on cons. Ile	0	4	2	0

**Table 2.** ATN codons in *Cob* in Oikopleuridae.

	*O. dio.*	*O. lon.*	*B. sty.*	*M. ery.*
ATA number	6	9	9	0
ATC number	5	2	1	2
ATG number	9	9	8	16
ATT number	21	11	11	22
ATA on cons. Met	0	0	0	0
ATA on cons. Ile	0	1	1	0

The TGA codon is known to encode tryptophan (Trp) in vertebrate, invertebrate and ascidian mitochondria ( Fox, 1979). We found that *B. stygius* uses TGA at positions where all other species would encode Arg ( Table 3 and Table 4). This is surprising as these two amino acids are unlikely to functionally substitute for each other. *O. longicauda* does not use TGA codons, and *M. erythrocephalus* does not use TGA at conserved Arg, although it is found at a position where all other species encode for Arg except *C. elegans* which encodes lysine, the other positively charged amino-acid. This again suggests a possible change of genetic code, although the numbers are currently too small to draw a solid conclusion.

**Table 3.** TGR codons in *Cox1* in Oikopleuridae.

	*O. dio.*	*O. lon.*	*B. sty.*	*M. ery.*
TGA number	2	0	3	1
TGG number	13	16	19	16
TGA on cons. Trp	1	0	0	0
TGA on cons. Arg	0	0	2	0

**Table 4.** TGR codons in *Cob* in Oikopleuridae.

	*O. dio.*	*O. lon.*	*B. sty.*	*M. ery.*
TGA number	3	0	2	1
TGG number	4	7	4	5
TGA on cons. Trp	1	0	0	0
TGA on cons. Arg	0	0	1	0

## Discussion

We extracted *Cox1* and *Cob* sequences of four different appendicularians from public databases. As a nucleotide sequence, *Cox1* might be useful for mining databases of molecular barcodes sequenced from the environment, or for studies of population diversity within a species. As a protein sequence, Cox1 might be useful for refining the phylogeny of appendicularians. However, a translation code needs to be chosen.

Our alignments of tunicate Cox1 and Cob protein sequences support the view that all tunicates translate AGR codons as Gly (although this conclusion might be limited by the lack of coverage for the Kowalevskiidae and Fritillariidae families). While our analysis suggests that the last common ancestor of the tunicates used the “ascidian” code, it is not possible to conclude that all contemporary tunicates still do, as we found discrepancies on other conserved residues that could be explained by a genetic code change of ATA and TGA codons within a sub-clade of the appendicularians containing *M. erythrocephalus*, *O. longicauda* and *B. stygius*.

The “ascidian” genetic code is table number 13 in the NCBI protein database, where it is used to translate sequences from ascidians and non-ascidian tunicates, for instance *D. nationalis*. However for appendicularians, the NCBI currently applies the invertebrate table (number 5). This has the consequences of turning Gly to Ser at functionally important positions. Therefore, the ascidian is probably a more appropriate default. At present, it is unclear whether some appendicularians have additional changes; however, the accurate translation of AGR codons to Gly would nonetheless reduce the amount of error in translated protein sequences.

To confirm a change of genetic code, it is necessary to detect corresponding changes in the respective tRNAs. This beyond reach for the present study because the mitochondrial genomic sequences that we used are extracted from draft genome sequences that may be incomplete, or even contain contaminations (see *B. stygius* in the Methods section). As a result, we also cannot entirely rule out the possibility that we have examined pseudogenes, although the high conservation found in the alignments suggest this in unlikely. For all these reasons, it is necessary to sequence full-length mitochondrial genomes from appendicularians.

## Conclusions

Our alignments of translated mitochondrial sequences suggest that the last common ancestor of living tunicates may have already used the “ascidian” genetic code. Thus, we recommend the use of that code instead of the “invertebrate” one for all tunicates in automatic translation pipelines, with the caveat that additional changes might be found in appendicularians. This observation is a reminder that in biology, exception is the rule, and that each time a mitochondrial sequence is extracted from a species for the first time, it is important to carefully examine its genetic code.

## Data availability

### Underlying data

All data underlying the results are available as part of the article and no additional source data are required.

### Extended data

Zenodo: Aligned Cox1 and Cob sequences from Oikopleura dioica and other tunicates. https://doi.org/10.5281/zenodo.3490310 ( Plessy & Pichon, 2019).

This project contains alignment files and descriptions of how the files were generated.

Extended data are available under the terms of the Creative Commons Zero “No rights reserved” data waiver (CC0 1.0 Public domain dedication).
