# Supplementary material for: Widespread use of the “ascidian” mitochondrial genetic code in tunicates
Source: F1000Res. 2020 Apr 14;8:2072. Originally published 2019 Dec 10. [Version 2] doi: 10.12688/f1000research.21551.2 (PMC7047913; doi:10.12688/f1000research.21551.2)
Supplement: Peer review history

# Peer review: Reviewer response for version 1

**DOI:** 10.5256/f1000research.23747.r59671

**Published:** 2020-02-26

**Authors:** Yuanning Li

**Affiliations:** 1 Department of Ecology and Evolutionary Biology, Yale University, New Haven, CT, USA

Pichon *et al.* conducted a study by comparing two mt genes across tunicates and recovered conserved tunicate-specific mt codon usage and potential Oikopleuridae-codon. The writing of the manuscript is clear and easy to follow. The methods are also valid and the findings should be interesting and important to the field. However, there are a few things I suggest to incorporate in the current draft.

1. In the introduction, the authors mainly discussed the tunicate genetic codon, but we already know there are more codon changes in deuterostomes (e.g. Hemichordates contain two mt genetic codons). So it would be better to incorporate this part of the introduction to make sure readers understand there are many mt codon changes within deuterostomes.
2. The authors also briefly discussed the possible change of tRNA structures responsible for codon change. Is it possible to extract tRNA sequences from available tunicate transcriptomic data and compare them to the existing ones? I am also fine with this if that is beyond the scope of this manuscript.

Is the work clearly and accurately presented and does it cite the current literature?

Yes

If applicable, is the statistical analysis and its interpretation appropriate?

Yes

Are all the source data underlying the results available to ensure full reproducibility?

Yes

Is the study design appropriate and is the work technically sound?

Yes

Are the conclusions drawn adequately supported by the results?

Yes

Are sufficient details of methods and analysis provided to allow replication by others?

Yes

Reviewer Expertise:

Evolutionary genomics and phylogenetics in marine invertebrates.

I confirm that I have read this submission and believe that I have an appropriate level of expertise to confirm that it is of an acceptable scientific standard.

# Response

**Published:** 2020-03-27

**Authors:** Charles Plessy

**Affiliations:** Okinawa Institute of Science and Technology Graduate University, Japan

We thank the reviewer for their 2 suggestions.

 1) “ *To make sure readers understand there are many mt codon changes within deuterostomes*”, we are adding the following text and references to our introduction:

 In animals, alternative genetic codes have first been found in large clades, for instance echinoderms (Himeno *et al.*, 1987) and hemichordates (Castresana *et al.*, 1998), but more recent works underline the presence of changes deeper in the phylogenetic tree, for instance within nematodes (Jacob *et al.*, 2009) and within hemichordates (Li *et al.*, 2019).

 2) On whether it is “ *possible to extract tRNA sequences from available tunicate transcriptomic data*”: as absence of evidence is not evidence for absence, our standpoint is that a rigorous analysis using a reference mitochondrial genome will be preferable. We hope that our methods section, that points at genomic scaffolds that are potential drafts of mitochondrial genomes, will be useful to the researchers interested in pursuing this direction.

# Peer review: Reviewer response for version 1

**DOI:** 10.5256/f1000research.23747.r57710

**Published:** 2020-01-13

**Authors:** Patrick Lemaire

**Affiliations:** 1 Montpellier Cell Biology Research Center (CRBM), CNRS, University of Montpellier, Montpellier, France

In this short article Pichon and colleagues use publicly available molecular datasets, mostly partial mitochondrial genomes, to re-explore the mitochondrial genetic code across tunicates, based on the analysis of the Cox1 and Cob genes.

 Their data suggest that the AGR codon was already translated into Glycine in the last common ancestor of tunicates and that additional changes may have occured in some Oikopleuridae at least. The work is important because it shows that all tunicates, including appendicularians should be associated in Genbank to the "ascidian" genetic code (Table 13).

 A limitation of the work, which the authors acknowledge, is that they could not identify the corresponding tRNAs in appendicularians. They therefore call for the sequencing of full-length mitochondrial genomes for appendicularians.

Is the work clearly and accurately presented and does it cite the current literature?

Yes

If applicable, is the statistical analysis and its interpretation appropriate?

Not applicable

Are all the source data underlying the results available to ensure full reproducibility?

Yes

Is the study design appropriate and is the work technically sound?

Yes

Are the conclusions drawn adequately supported by the results?

Yes

Are sufficient details of methods and analysis provided to allow replication by others?

Yes

Reviewer Expertise:

Tunicate embryology and genomics

I confirm that I have read this submission and believe that I have an appropriate level of expertise to confirm that it is of an acceptable scientific standard.
